# Signals of increasing co-use of stimulants and opioids from online drug forum data

**DOI:** 10.1186/s12954-022-00628-2

**Published:** 2022-05-25

**Authors:** Abeed Sarker, Mohammed Ali Al-Garadi, Yao Ge, Nisha Nataraj, Christopher M. Jones, Steven A. Sumner

**Affiliations:** 1 Department of Biomedical Informatics, School of Medicine, Emory University, 101 Woodruff Circle, Suite 4101, Atlanta, GA 30322 USA; 2 National Center for Injury Prevention and Control, Centers for Disease Control and Prevention, Atlanta, GA 30341 USA

**Keywords:** Opioids, Stimulants, Substance use, Natural language processing, Social media

## Abstract

**Background:**

Despite recent rises in fatal overdoses involving multiple substances, there is a paucity of knowledge about stimulant co-use patterns among people who use opioids (PWUO) or people being treated with medications for opioid use disorder (PTMOUD). A better understanding of the timing and patterns in stimulant co-use among PWUO based on mentions of these substances on social media can help inform prevention programs, policy, and future research directions. This study examines stimulant co-mention trends among PWUO/PTMOUD on social media over multiple years.

**Methods:**

We collected publicly available data from 14 forums on Reddit (subreddits) that focused on prescription and illicit opioids, and medications for opioid use disorder (MOUD). Collected data ranged from 2011 to 2020, and we also collected timelines comprising past posts from a sample of Reddit users (Redditors) on these forums. We applied natural language processing to generate lexical variants of all included prescription and illicit opioids and stimulants and detect mentions of them on the chosen subreddits. Finally, we analyzed and described trends and patterns in co-mentions.

**Results:**

Posts collected for 13,812 Redditors showed that 12,306 (89.1%) mentioned at least 1 opioid, opioid-related medication, or stimulant. Analyses revealed that the number and proportion of Redditors mentioning both opioids and/or opioid-related medications and stimulants steadily increased over time. Relative rates of co-mentions by the same Redditor of heroin and methamphetamine, the substances most commonly co-mentioned, decreased in recent years, while co-mentions of both fentanyl and MOUD with methamphetamine increased.

**Conclusion:**

Our analyses reflect increasing mentions of stimulants, particularly methamphetamine, among PWUO/PTMOUD, which closely resembles the growth in overdose deaths involving both opioids and stimulants. These findings are consistent with recent reports suggesting increasing stimulant use among people receiving treatment for opioid use disorder. These data offer insights on emerging trends in the overdose epidemic and underscore the importance of scaling efforts to address co-occurring opioid and stimulant use including harm reduction and comprehensive healthcare access spanning mental-health services and substance use disorder treatment.

**Supplementary Information:**

The online version contains supplementary material available at 10.1186/s12954-022-00628-2.

## Background

Stimulant co-use [1–4], specifically methamphetamine, and stimulant co-involvement in overdose fatalities [4], including among people with opioid use disorder (OUD), are of growing concern in the USA and around the world. This increase in co-use is likely contributing to statistically significant increases in overdose deaths involving both opioids and stimulants [5]. The rise in overdose deaths involving opioids and stimulants has exacerbated the challenge of addressing the decades-long opioid overdose crisis and reflects the evolving, polysubstance nature of the broader overdose epidemic and the presence of illicitly manufactured fentanyl [6]. Studies have shown that stimulant co-use exposes people who use opioids (PWUO) to additional health risks, including overdose, infectious disease transmission, and suboptimal treatment outcomes [7, 8]. Further, these changes in substance use patterns and related harms have occurred against the backdrop of a substantial increase in availability of methamphetamine across the globe and the USA. In the USA, based on drug product submissions to the Drug Enforcement Administration’s National Forensic Laboratory Information System, methamphetamine submissions more than doubled from 2011 to 2019 [9]. In addition, in the 2020 National Drug Threat Assessment, methamphetamine seizures and price data as well as law enforcement reporting all indicate that methamphetamine is now readily available at a greater purity and potency throughout the USA [10].

Recent findings also suggest that reasons for co-use, particularly with methamphetamine, are multifaceted—such as ease of access, helping boost the “high,” improving daily function (e.g., increasing productivity or staying alert), and helping manage withdrawal symptoms [11]. However, little is known about when stimulants enter into the substance use trajectory of PWUO, how co-use patterns have shifted over recent years, and how patterns of opioid and stimulant co-use evolve at an individual level. One potential source for gathering exploratory information on this emerging health concern is longitudinal social media data obtained from Reddit. Reddit data are being increasingly used for conducting observational research, particularly on sensitive topics such as substance use [12–15].

### Objectives

The overarching objectives of this study are to utilize social media conversation data on Reddit to:

1. Estimate the volumes and short- and long-term trends of opioid-stimulant co-mentions
2. Quantify estimated co-use rates between pairs of specific opioids and stimulants based on these discussions
3. Identify when stimulants are initiated evidenced by mentions among PWUO at an individual level based on timelines of posts

The insights we derive in this study are purely data-driven through natural language processing (NLP) methods, as opposed to hypothesis-driven, and our methods account for growth in the number of Reddit users over time. Being data-driven, we did not start our analyses with any underlying assumptions or hypothesis. Our intuition was that the growing number of overdose deaths due to co-use of opioids and stimulants would be reflected on the Reddit data, but this intuition did not affect our approach. A better understanding of the timing and patterns in stimulant co-use among PWUO as evidenced by mentions of these substances on social media can help inform prevention efforts including harm reduction, as well as future research directions. In the remainder of the article, we refer to people who post on specific subreddits of interest as Redditors, people who use or used prescription and/or illicit opioids, people who used an opioid reversal agent (e.g., naloxone), and people with OUD who are being treated with medications for opioid use disorder (MOUD) as PWUO/PTMOUD, people who use stimulants as PWUS, people who use drugs more broadly as PWUD, and people who co-use opioids and stimulants as PWCU.

## Methods

### Data

Reddit is particularly popular among PWUO and the broader community of PWUD as it can offer anonymity [16]. Reddit has seen rapid growth in its user base over the last several years. In 2021, it is estimated that Reddit has over 430 million [17] monthly active users (Redditors), surpassing the number of active Twitter users [18]. These increases may be driven by factors such as the organization of discussions through specialized topical-interest forums called *subreddits* and the moderation of forum-specific content by forum users. Reddit communities have also been found to serve as a means of social support for PWUD [14, 15, 19]. We chose Reddit over other social networks or web-based forums such as Twitter [20], Bluelight [21], and Discord [22] for several reasons. While all these sources contain information about substance use, the substance use community of Reddit is much larger and has been extensively used in peer-reviewed research related to substance use and emerging substance use trends [23–25]. Reddit content is also moderated, and posts that do not adhere to the rules of a subreddit are removed by its moderators. Consequently, while these rules restrict some types of information from being posted, they also ensure that the data are reflective of the topical areas and the volume of spam, posts from bots, or irrelevant content is thereby lower. The existence of standard application programming interfaces (APIs) also makes data collection from Reddit relatively straightforward.

To find potential PWUO on Reddit, we identified 14 opioid-related subreddits spanning discussions on prescription and illicit opioids and MOUD (Additional file 1: Appendix A1) and collected all retrievable posts contained using the Python-Reddit API Wrapper for Reddit [26]. The subreddits were chosen by clinical domain expert coauthors (SAS and CMJ). The choice of these subreddits was based on their topical relevance and high levels of community discussion and engagement. Collection of data from these subreddits was not keyword based. Instead, the API allowed the retrieval of all publicly posted threads and the associated comments. The API did not enable us to retrieve posts that were either removed by the moderators or the original posters. Thus, subscribers who intended their posts to no longer be accessible were not included in the study. Since these subreddits serve as specific discussion forums for topics related to opioid use and recovery and empirical examination of posts revealed that most Redditors discussed personal use and experience, we assumed that all authors of posts that mentioned any opioid or opioid-related medications defined as opioid reversal agents and MOUD were either current or past PWUO/PTMOUD. We also assumed that mentions of a substance corresponded with the presumed use of that substance. Thus, Redditors who mentioned opioids and/or opioid-related medications and stimulants were presumed to co-use both substances (PWCU) during the study period—in our analyses, we separately examined co-mentions of both substances within a given year as well as mentions that may have spanned multiple years. After retrieving all available posts of the 47,327 Redditors who had posted on the selected subreddits, we selected a random sample of these Redditors (N = 13,812) and collected each of their past public posts across all subreddits (i.e., timelines), between November 2006 (corresponding to the earliest post available) and July 2021 (corresponding to the last date of data collection). Due to the restrictions of the API, all posts by an individual subscriber could only be retrieved by traversing through all the subreddits on which they posted. Each timeline took approximately 20 min to obtain, so the number of Redditors included in the sample was limited by the rate at which we could collect full timelines via the API. We studied trends in opioid and stimulant co-use between January 1, 2011 and December 31, 2020. We excluded earlier years from this analysis because the numbers of posts (and correspondingly, Redditors) prior to 2011 were very low (i.e., ≤ 20 posts), and we excluded 2021 because complete data for this year were not available. Due to similar concerns related to low number of posts with specific substances mentioned, we restricted our study of specific opioid and opioid-related medications, and stimulant categories to the years 2015–2020. We then chronologically ordered the posts per Redditor to enable an exploration of substance use timelines. All analyses described in this study were conducted using Python.

### Identifying substance mentions

We applied natural language processing methods to improve the detection of true mentions of opioids and stimulants and to exclude false positives (e.g., negated concepts and ambiguous expressions). We focused the study on a set of common opioids, opioid-related medications, and stimulants, including both prescription and illicit types (Additional file 1: Appendix A2). We decided to include substances that contained mixtures of stimulants and opioids (such as a combination of methamphetamine and heroin, i.e., “goofball”/“speedball”) under the stimulant category. This choice was based on the fact that our overarching objective was to study stimulant co-use trends among PWUOs, and so people describing the use of these mixed substances on opioid subreddits were likely to be indicating co-use.

The application of NLP primarily focused on ensuring that we detect large numbers of mentions of the substances and removing potential false positives automatically. The first application of NLP was to generate lexical variants (e.g., misspellings) of the substances. Since drug names and related expressions are often misspelled on social media, we generated commonly used lexical variants of the terms using the LexExp tool [27]. We found that some non-standard terms and lexical variants tend to have high noise associated with them (i.e., expressions not actually referring to a stimulant or opioid, e.g., *oxy clean)*. Thus, we next included additional filters for the terms *stimulant*, *meth*, and *oxy* (Additional file 1: Appendix A3). This NLP method enabled us to filter out many false positives.

The third NLP task involved implementing a negation detection strategy, customized for Reddit data, to exclude negated drug mentions. Specifically, we used a subset of negation terms from the NegEx algorithm [28] (Additional file 1: Appendix A4) with a moving context window, determined empirically, of size *n* = 5 following the mention of a negation trigger. Any opioid, opioid-related medication or stimulant term was considered negated if it appeared in the context window following a detected negation expression and if an end of sentence marker (*e.g*., a period) did not occur between the negation term and the drug term. Negated terms were excluded from the final count of mentions. We also manually reviewed samples of posts detected by our searches to identify keywords with a large amount of noise (e.g., “*dope*” for heroin and “*coke”* for cocaine) and excluded those from our counts and further analysis. Of the 13,812 Redditors sampled, a subset who posted in these subreddits had no mention of either an opioid, opioid-related medication or a stimulant (N = 1506) and was excluded from the analyses. All analyses except for those examining the total numbers of Redditors (including PWCU) over time were based on subsets of the remaining 12,306 Redditors who posted about at least one opioid, opioid-related medication, or stimulant.

### Opioid and stimulant co-use trends

We first studied trends in opioid and stimulant co-use between 2011 and 2020 through multiple metrics, including, the number of Redditors posting each year; the number of Redditors who mentioned at least one opioid or opioid-related medication and one stimulant (*i.e.*, PWCU) over the same time frame; and the annual ratio of PWCU to PWUO/PTMOUD. Due to the growing user base of Reddit over time, we expected numbers of PWUO/PTMOUD and PWCU to both increase annually. Hence, the ratio of PWCU to PWUO/PTMOUD likely serves as the best indicator of social media trends in opioid and stimulant co-use within this population.

Next, we calculated and visualized three main explorations of co-use patterns for our dataset, focusing on the years 2015–2020 due to lower counts of posts by specific substances in prior years,—(1) frequency of co-use of specific types of opioid-stimulant pairs among PWUO/PTMOUD aggregated over the entire study period; (2) proportion of specific opioids co-used with any stimulant in a given year among the total number of PWCU in that year, to study how mentions of specific opioids or opioid-related medications co-used with any stimulant changed over time; and (3) proportion of specific stimulants co-used with any opioid or opioid-related medication in a given year among the total number of PWCU in that year, to study how mentions of specific stimulants co-used with any opioid changed over time. Redditors with mentions of more than one type of opioid or opioid-related medication or stimulant within a given year were separately considered for each specific opioid-stimulant pair.

We combined the opioids and opioid-related medications listed in our analytic sample into 5 categories: (1) heroin, (2) fentanyl & analogs (including carfentanil), (3) Medications for Opioid Use Disorder (MOUD, e.g., buprenorphine, methadone, naltrexone), (4) opioid overdose reversal agents (e.g., naloxone), and (5) prescription opioid pain relievers (e.g., oxycodone, hydrocodone, and tramadol). We combined stimulants into 4 categories: (1) methamphetamine, (2) amphetamine-type stimulants (e.g., Adderall®, dextroamphetamine, levoamphetamine), (3) methylphenidate-type stimulants (e.g., Ritalin®), and (4) a combination of methamphetamine and heroin (i.e., “goofball”/”speedball”). Categories included generic names, trade names, and common misspellings or lexical variants (Additional file 1: Appendix A2). Due to the aforementioned challenges of appropriately identifying keywords with substantial noise, we excluded mentions of cocaine from our analysis.

### Timeline analyses

One of our objectives was to explore when stimulants are initiated and used by PWUO/PTMOUD over time. We focused specifically on methamphetamine initiation and use as our analyses revealed methamphetamine to be the most commonly used stimulant by a large margin (Additional file 1: Appendix A5). We constructed a cohort by selecting the subset of Redditors who mentioned an opioid or opioid-related medication first in their timelines of posts from our randomly selected sample. To align the timelines of different Redditors, we considered the date of the first opioid mention to be Day 0. Timelines were stratified based on opioid type by grouping together Redditors who mentioned the same or similar opioids on Day 0. We tracked timelines for each PWUO/PTMOUD by computing the monthly frequencies of methamphetamine mentions for 24 months starting from Day 0. We excluded Redditors whose first and last posts on Reddit were less than 24 months apart and those who had any stimulant-related posts within 30 days of their first opioid or opioid-related medication post (i.e., individuals with presumed co-use within the first month). By mapping the first post to Day 0 for all Redditors who posted, the distribution of posts over the 24 months was naturally skewed—each Redditor had at least one post in the first month, but the frequency of posts over the following months varied. To adjust for this, we normalized the monthly methamphetamine mention frequencies by the total posts made by PWUO/PTMOUD each month. Since most mentions correspond to personal use, we assumed Redditors with an opioid or opioid-related medication mention first and subsequent methamphetamine mention had transitioned to co-use. We developed heatmaps of these timelines to visualize temporal patterns of methamphetamine use.

## Results

### Study sample characteristics

We collected 13,812 Redditor timelines from the 14 subreddits, 12,306 of whom mentioned at least one of our selected opioid, opioid-related medication, or stimulant keywords. Redditors mentioning any opioid or opioid-related medication, or any stimulant in their timelines were approximately evenly distributed—9329 Redditors mentioned opioids or opioid-related medication at some point in their timeline; 9151 mentioned stimulants (Additional file 1: Appendix A5). There was a substantial proportion of presumed PWCU among Redditors who mentioned at least one opioid, opioid-related medication, or stimulant keyword—6174 (50.2%) Redditors mentioned both an opioid or opioid-related medication and a stimulant. However, there was a large difference in the *first* substance mentioned—almost twice as many Redditors mentioned an opioid or opioid-related medication first in their timelines (*n* = 8276/12,306; 67.3%) versus those who mentioned a stimulant first (*n* = 4012/12,306; 32.6%).

### Co-use characteristics

**Fig. 1:**
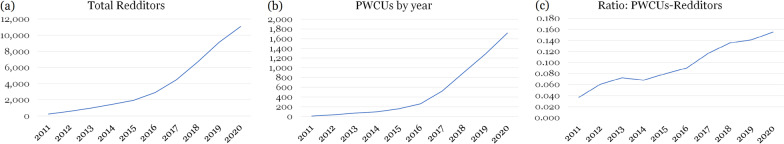
**a** Total number of Redditors who posted in the opioid-related subreddits each year from 2011 to 2020; **b** total number of people who co-use opioids and stimulants (PWCU), i.e., who posted about both opioids and/or opioid-related medications and stimulants; and **c** ratio of PWCU to total number of Redditors each year. Volume of participation in relevant subreddits in early years of the analysis is low; caution in interpreting trend should be applied

Figure 1 shows the (a) total number of Redditors per year posting in the 14 opioid-related subreddits, (b) number of PWCU per year as defined by the number of persons posting about both opioids and stimulants within the same year, and (c) ratio of PWCU to total Redditors per year from 2011 to 2020 in our analytic sample. These metrics were computed independently each year, i.e., a PWUO/PTMOUD or PWCU was determined based only on the posts within that year. (Additional file 1: Appendix A6 provides additional metrics.) Figure 1a, b illustrates that the total number of Redditors posting on opioid-related subreddits (including Redditors with and without mentions of opioid or stimulant keywords of interest) as well as those posting about both opioids or opioid-related medications and stimulants (PWCU) increased each year since 2011, with sharp increases observed after 2016. The proportion of individuals posting about both opioids or opioid-related medications and stimulants as a fraction of all individuals posting also showed consistent annual growth (Fig. 1c). By 2020, more than 15% of individuals posting in opioid-related subreddits had also posted about a stimulant.

**Fig. 2:**
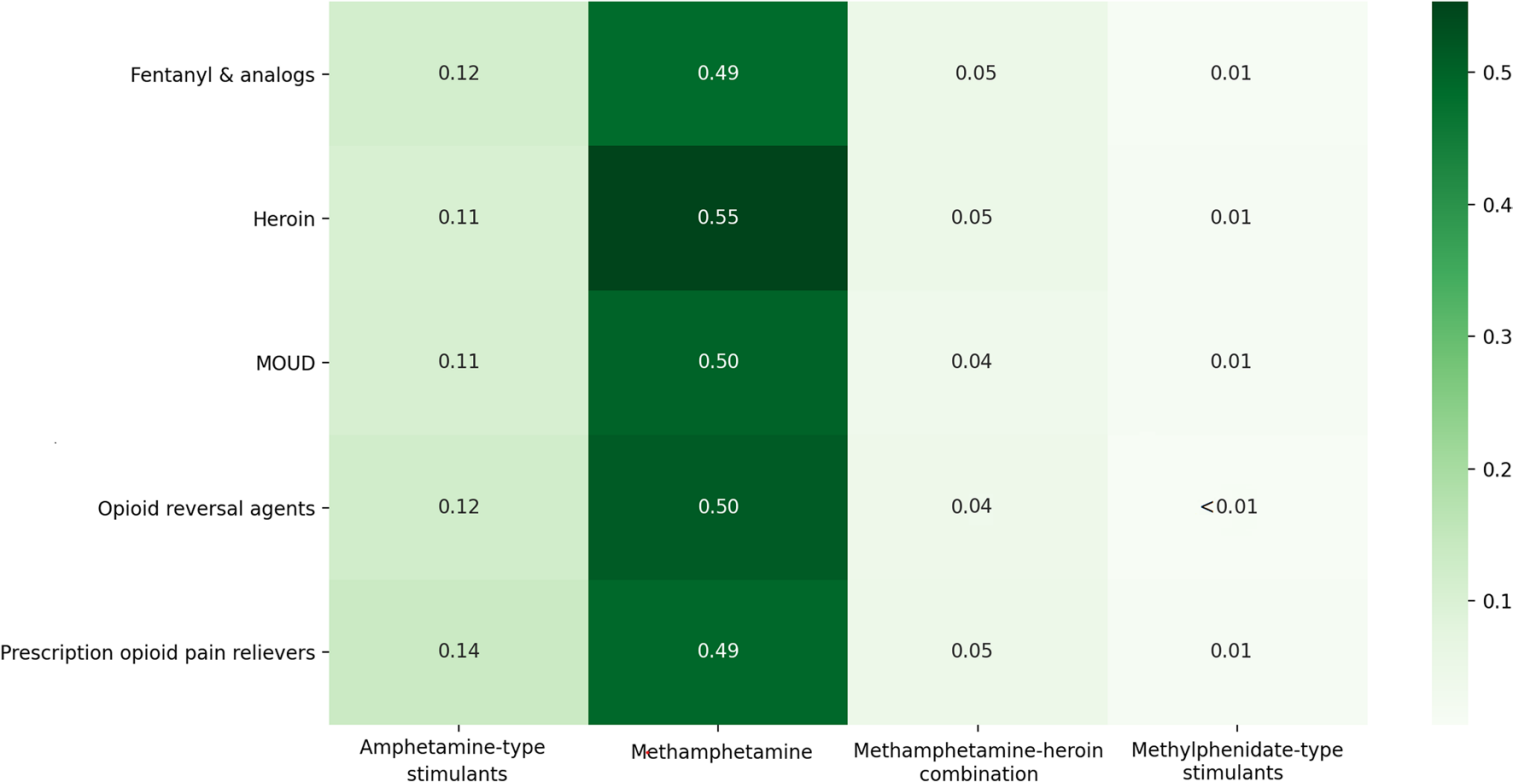
Proportions of Redditors who posted about specific opioid and opioid-related medication and stimulant pairs (e.g., opioid *i* and stimulant *j*) among all Redditors who posted about each specific opioid or opioid-related medication (e.g., opioid *i*). Opioids, opioid-related medication, and stimulants are grouped as described in “Opioid and stimulant co-use trends” section. Total number of Redditors who mentioned at least one opioid = 9329. Total number of Redditors who mentioned at least 1 stimulant = 9151

Figure 2 shows heatmaps of the proportion reporting opioid and stimulant co-use among PWUO/PTMOUD. Within our sample, co-mentions of heroin and methamphetamine were most commonly observed relative to other opioid and opioid-related medications and stimulant types across 2011–2020. Indeed, across all opioids and opioid-related medications, methamphetamine was the most frequently co-mentioned stimulant. Amphetamine-type stimulants appeared to have the highest co-mentions after methamphetamine, followed by methamphetamine-heroin combinations (“speedball”/ “goofball”). The relatively large volume of mentions of amphetamine-type stimulants was primarily driven by discussions of Adderall®, a prescription stimulant (Additional file 1: Appendix A5). Interestingly, amphetamine-type stimulants appeared to have the highest probability of co-use with prescription opioid pain relievers (e.g., hydrocodone and oxycodone) relative to illicit opioids such as heroin.

### Co-use over years

**Fig. 3:**
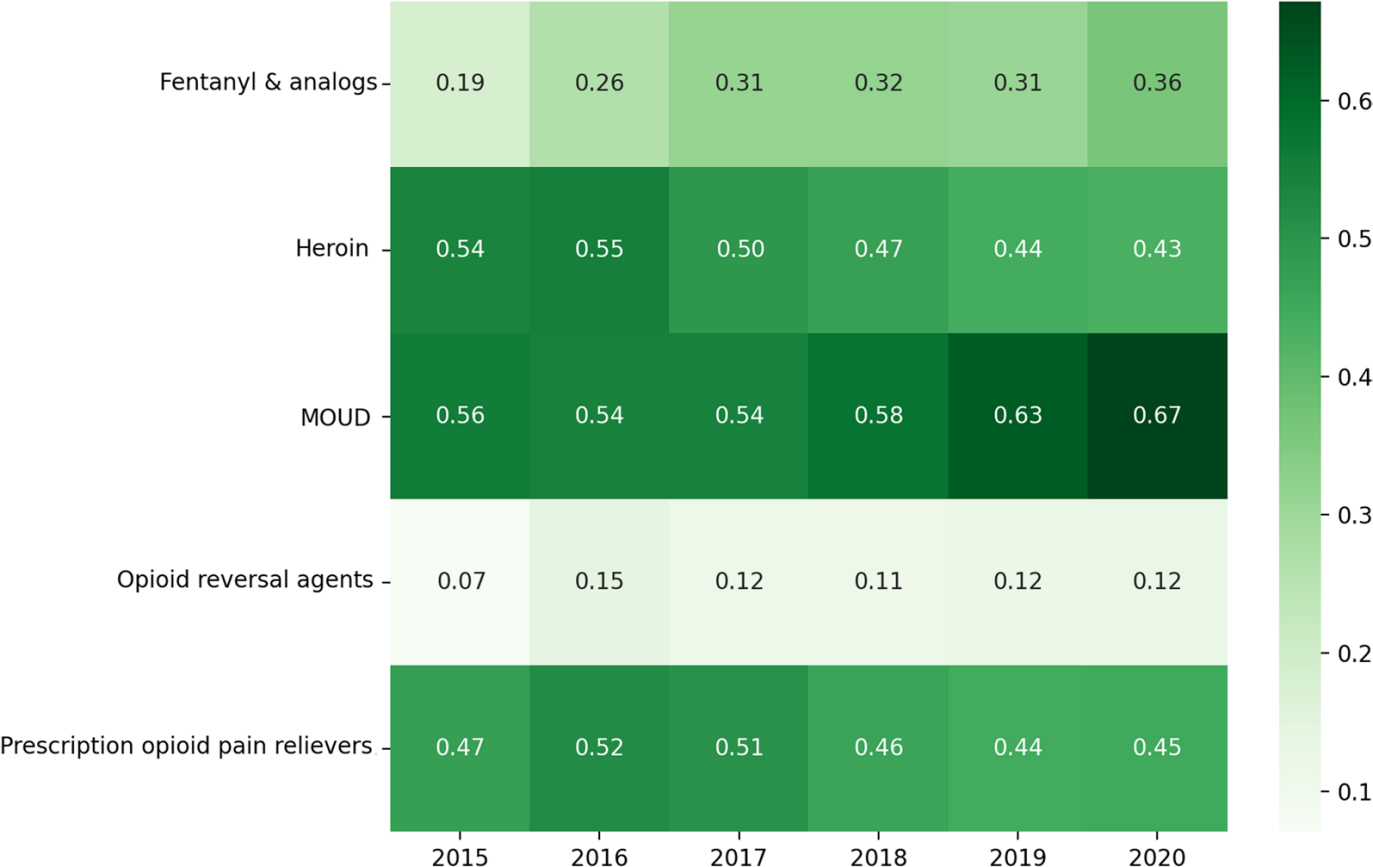
Proportions of people who co-use opioids and stimulants (PWCU, *N* = 4786) who mentioned co-use of specific groups of opioids or opioid-related medications with any stimulant by year from 2015 to 2020. Total number of unique people who use opioids (PWUO)/people with OUD who are being treated with medications for opioid use disorder (PTMOUD) within this timeframe = 8831. Opioid-related categories—Fentanyl and analogs (fentanyl and carfentanil); Heroin; Medications for opioid use disorder (MOUD: suboxone, sublocade, methadone, buprenorphine, naltrexone); Opioid reversal agents (Naloxone); and Prescription opioid pain relievers (hydrocodone, oxycodone, morphine, tramadol)

Figure 3 shows the proportion of individuals who posted about particular opioid-related categories among those posting about both opioids and stimulants within a given year. This helps illustrate which opioid-related categories were most commonly mentioned among PWCU and how mentions of these opioids and opioid-related medications in the setting of co-use have potentially changed over time—e.g., fentanyl and its analogs had a relatively low probability of being discussed with stimulants in 2015, but nearly doubled by 2020. Mentions of MOUD similarly increased, while those of heroin decreased over time among PWCU.

**Fig. 4:**
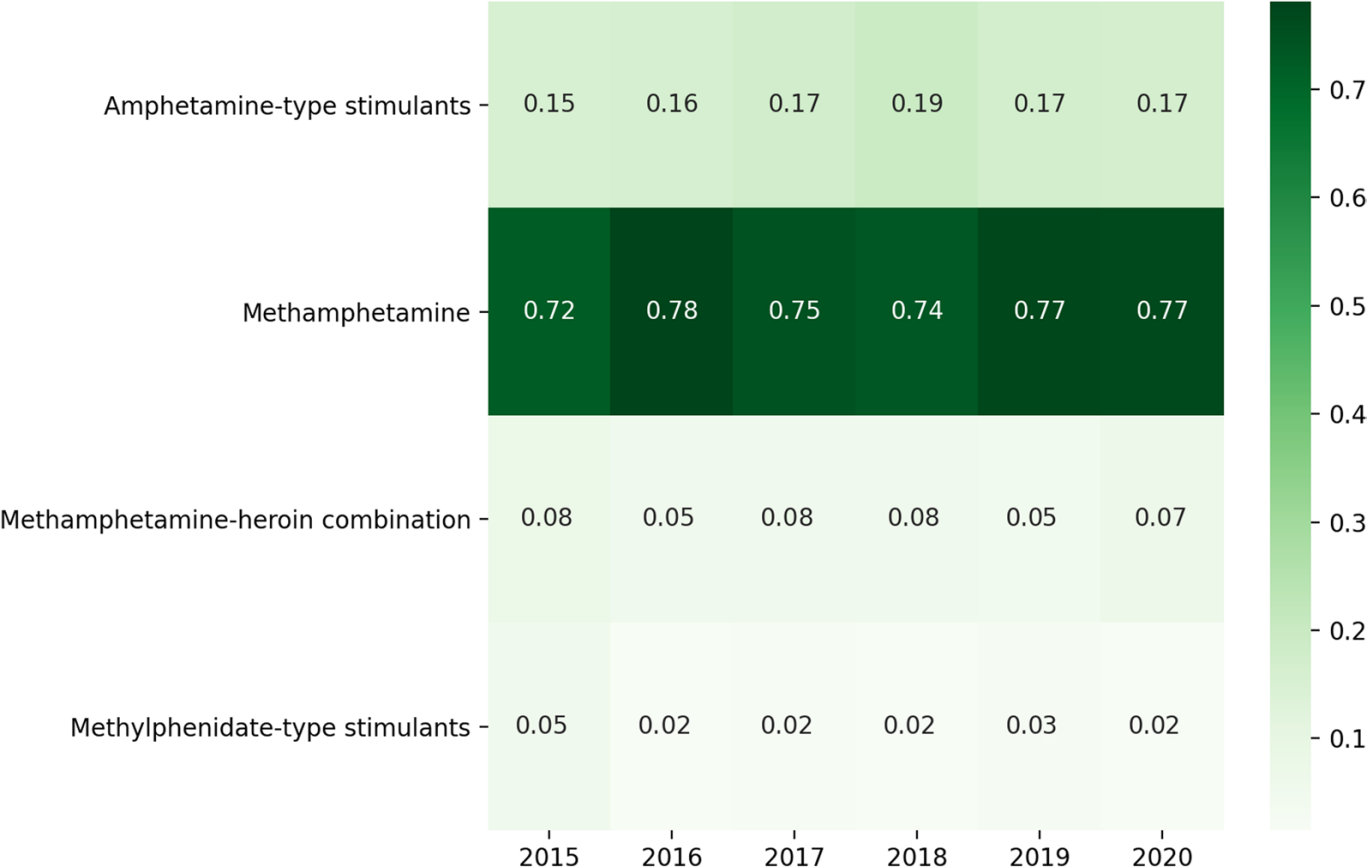
Proportions of people who mentioned co-use of specific groups of stimulants with any opioid or opioid-related medication (PWCU, *N* = 4786) by year from 2015 to 2020. Total number of people who use stimulants (PWUS) within this timeframe = 8266. Stimulant categories—Amphetamine-type stimulants (amphetamine, dextroamphetamine, levoamphetamine, and lisdexamphetamine); Methamphetamine; Methylphenidate-type stimulants (methylphenidate); and a combination of methamphetamine and heroin (“speedball”/”goofball”)

Figure 4 shows the proportion of individuals posting about a particular stimulant among all individuals posting about both opioids or opioid-related medications and stimulants that same year. Methamphetamine mentions largely eclipsed all other stimulants studied. Amphetamine-type stimulants showed the next highest co-use and increase over time.

### Timeline analysis results

**Fig. 5:**
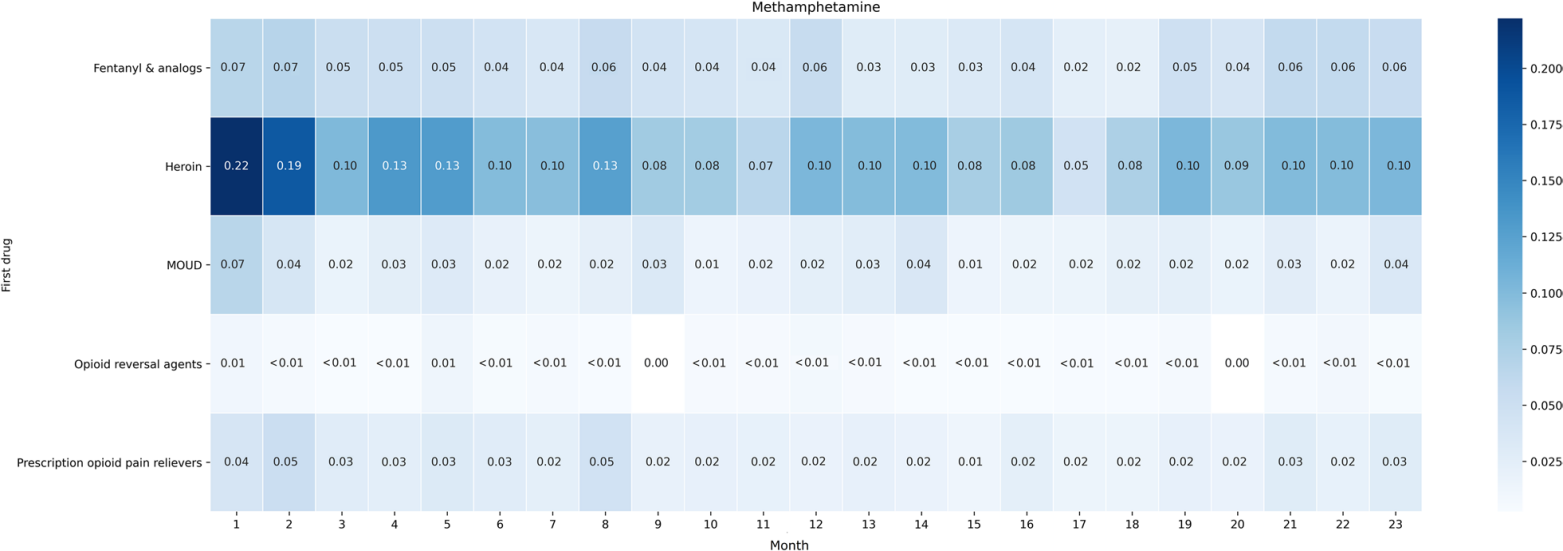
24-month (excluding the first month) monthly relative frequencies of methamphetamine mentions among Redditors who first mentioned an opioid or opioid-related medication (N = 849). Mention frequencies per month are normalized by the total number of posts by the same set of Redditors within that specific time span. Opioid-related categories—Fentanyl and analogs (fentanyl and carfentanil); Heroin; Medications for opioid use disorder (MOUD: suboxone, sublocade, methadone, buprenorphine, naltrexone); Opioid reversal agents (Naloxone); and Prescription opioid pain relievers (hydrocodone, oxycodone, morphine, tramadol). Stimulant categories—Amphetamine-type stimulants (amphetamine, dextroamphetamine, levoamphetamine, and lisdexamphetamine); Methamphetamine; Methylphenidate-type stimulants (methylphenidate); and a combination of methamphetamine and heroin (“speedball”/”goofball”)

Figure 5 illustrates the initiation of and patterns in methamphetamine mentions among the cohort of individuals who first mentioned opioids. Overall, 849 Redditors from our sample met the inclusion criteria (i.e., had posts spanning > 24 months, first mentioned an opioid or opioid-related medication, and no stimulant posts prior to or < 30 days of first opioid post) with a total of 4,303 posts. Specifically, our figures show 24-month heatmaps where each month displays the relative frequencies of methamphetamine mentions normalized by the total number of posts made by PWUO/PTMOUD in that month. The heatmaps are stratified by the first opioid or opioid-related medication type the individual posted about in their timeline.

There are observable differences between the heatmaps associated with the different types of opioids. The heatmap for *heroin* suggests co-use discussions with methamphetamine occurred most frequently relatively early in PWCU timelines. Conversely, the *fentanyl  and analogs* heatmap shows a different pattern—methamphetamine co-use for these individuals demonstrates periods with elevated discussions at the beginning, middle, and end of the 24-month timeline. Heatmaps for *prescription opioid pain relievers* and *MOUD* were similar, with higher methamphetamine mentions at the beginning of the timelines and decreases toward the end; however, these decreases in mentions were smaller in magnitude relative to decreases among individuals who initially discussed heroin.

## Discussion

The findings from our analysis of social media data are consistent with those from traditional health data sources indicating increased opioid and stimulant co-use over time [3, 7], while also offering unique exploratory insights. While the opioid overdose epidemic has been ongoing for over a decade now, the surge in stimulant use, in particular methamphetamine use, among PWUO is a relatively recent public health concern. Several hypotheses have been suggested to explain this growing trend, including efforts to improve prescribing practices to reduce the availability of opioids, which may have resulted in shifts to other substances by PWUO [3].

When examining the most frequently co-mentioned substances between 2011 and 2020, we found that illicit opioids were more frequently mentioned with illicit stimulants (e.g., heroin and methamphetamine) and prescription opioids were most frequently mentioned with prescription amphetamine-type stimulants (e.g., oxycodone/hydrocodone and Adderall®). This suggests possibly distinct populations within the social media substance use forums we studied with preferences for particular types of substances. Further understanding the distinction between populations focused primarily on prescription drug misuse and those focused on illicit substance use, and the transitions between these preferences, is a future area for exploration of online data that may yield insights for better targeting prevention and harm reduction efforts. Indeed, while there is research on prescription stimulant misuse [24], less is known about its relationship to substance use trajectories, especially among individuals who misuse prescription opioids [29, 30].

We found noticeable increases in the proportion of stimulant mentions among individuals also discussing MOUD over time, consistent with recent reports indicating that stimulant use among people receiving treatment for OUD may be increasing [2, 3]. Co-use of stimulants among people with OUD is concerning given prior research demonstrating increased risk for health harms such as overdose as well as suboptimal treatment outcomes, including poorer retention in MOUD, among people with OUD who use stimulants [31,32,]. These findings together underscore the importance of scaling efforts to address co-occurring opioid and stimulant use through expansion of MOUD in combination with non-pharmacological treatment modalities that address stimulant use such as contingency management and community reinforcement approach or cognitive behavioral therapy, along with recovery support services [33].

While relative rates of stimulant co-use among individuals using fentanyl appear to be on the rise, the relative rate for co-use among individuals using heroin showed a gradual decrease. This is perhaps a result of changes in the illicit opioid supply over recent years, as heroin has experienced supply shortages [34, 35] and the illicit drug market in many communities has transitioned from a heroin-based market to a fentanyl-based market [35]. For persons using stimulants to counteract the depressant-type effects of opioids [36], the stronger potency of fentanyl may also be driving increased interest in stimulant use. Recent literature also suggests that PWUO/PTMOUD who knowingly or unknowingly consume fentanyl tend to have more severe withdrawal symptoms when taking MOUD [37], which may partially explain the greater interest over time in stimulants among Redditors discussing both MOUD and fentanyl. Our results suggest that as availability and use of synthetic opioids such as fentanyl continue to increase, interest in and co-use of stimulants could persist. Individuals with polysubstance use have unique treatment needs and have higher risks for mental health comorbidities [38], further emphasizing the need for increased availability of comprehensive healthcare including mental health services and substance use disorder treatment for PWUD. Qualitative research is needed to help better characterize the timings and concerns associated with initiation of co-use among PWUO in order to develop and appropriately direct prevention and response strategies that can address the needs of this population. Future research could qualitatively examine discussions on these forums to identify how co-use manifests and whether the contexts of these mentions has changed over time. These research directions can help better understand risks and motivations associated with polysubstance use, such as changes in the drug supply or other factors, and inform prevention strategies, including a move toward comprehensive community-level harm-reduction efforts [39].

Future work can also focus on further utilizing NLP and machine learning methodology to conduct more fine-grained characterization of the data, such as annotation of posts to filter out posts that do not self-report consumption of a substance mentioned [40–43]. Advanced NLP methods, such as those for relation extraction, can be applied to study the associations between opioids and stimulants in an individual’s social media timeline, and to study the contexts of co-use. The aggregated findings can be provided to the subject matter experts for validation.

### Limitations

Our study has several limitations. Absent contextual information, the primary limitation is the assumption that an individual mentioning an opioid, opioid-related medication, or stimulant is currently using that substance, which may result in overestimation of the number of PWUO/PTMOUD, PWUS, and PWCU, particularly in analyses where these mentions spanned multiple years. Nonetheless, the number of mentions could be a proxy for use and at minimum, mentions of a substance represent some degree of interest or consideration of a particular substance. Also, since the forums from which the Redditors were initially identified are topic-specific, most posts explicitly or implicitly refer to personal experiences.

Second, individuals who post on Reddit may not necessarily be representative of all PWUD and caution when generalizing from these findings is warranted. According to a 2019 PEW Research Center report, Reddit has an overrepresentation of males and younger people [44]. Since then, however, the user base or Reddit has grown significantly. Our paper primarily focuses on the overdose crisis in the USA although Reddit is a global platform. However, the vast majority of Reddit subscribers (~ 50%) are estimated to be from the USA, followed by the UK (~ 8%) and Canada (~ 8%) [45]. Thus, estimates derived from Reddit do indeed reflect characteristics of the US population better than any other country.

Third, not all PWUO, PWUS, and PWCU necessarily mention all the substances they may use. Additionally, we excluded cocaine from our analyses due to concerns around data quality given challenges associated with accurately identifying mentions. Further, our analyses were limited to opioid-related subreddits and excluded any stimulant-related subreddits given our focus on PWUO/PTMOUDs. These limitations may both lead to an underestimation of stimulant and opioid-stimulant co-use. Fourth, we were only able to conduct our analyses on a sample of Redditors due to the time required for retrieving longitudinal Redditor data. Our concerns about this limitation are mitigated by the finding that statistics derived from smaller samples of Redditors early on in our study remained almost identical to those obtained when larger numbers of individuals were included. The exponentially rising number of Reddit subscribers over time also posed a challenge for us, since we could not quantify how much of the increasing opioid or opioid-related medications and stimulant mentions could be attributed to increases in their use in the community versus the growing Redditor numbers. To address this, we examined the ratio of PWCUs to Redditors, which revealed that the growth rate in substance mentions was higher than the growth rate of Redditors. It is possible that other factors contributed to the rising substance mention rates, but we were unable to include such confounding factors in this analysis. Lastly, methodological limitations inherent to all analyses of unstructured text data apply as not all mentions/expressions are detectable by NLP methods [46, 47]. However, we employ robust and well-validated approaches for capturing spelling variants and non-standard expressions.

### Ethical considerations

Our study included only publicly available data that were retrievable via the Reddit API. No private information was obtained. Subreddits that require membership before viewing content were not included. Since our study falls under the category of observational studies conducted on publicly available data, the Emory University Institutional Review Board (IRB) deemed the study to be exempt (category 4). It was determined that no additional harm to individuals was introduced by the use of the publicly available data. In clinical settings, when protected clinical data of patients are used for research, the standard practice is to seek informed consent prior to inclusion. With social media based observational studies such as ours, obtaining consent requires contacting the subscribers directly, and this action requires a higher level of IRB approval as the direct communication with the subscriber may be considered to be intervention. Such contact also presents bigger challenges in determining if the study presents any additional risk or unanticipated harms to the individual. The ethical considerations that need to be taken into account when conducting research using social media data are evolving through discussions and collaborations among active researchers in this space. Currently, there is some consensus that it is appropriate, and perhaps necessary, to utilize all available public data for addressing serious public health crises, such as the ongoing opioid overdose epidemic [48]. Excluding this vast source of knowledge can be seen as a missed opportunity to identify potential prevention strategies. Our study is justified based on these factors, and we followed the accepted best practices used in many similar papers from the research community [15, 49–56.

## Conclusion

We examined real-world, longitudinal social media data from online opioid-related forums to explore patterns in opioid and stimulant discussions and found large increases in co-mentions of opioids or opioid-related medications and stimulants. These findings are consistent with those from other health data sources suggesting growing co-use of these substances and resultant harms. Although a multitude of factors may influence substance use trends, many are difficult to elucidate and explore using traditional data sources such as survey records or health care data. The social media data we explored offer important exploratory insights into which opioids or opioid-related medications and stimulants are most frequently co-mentioned and how these patterns have changed over time. These data derived from real-world conversations may help in hypothesis generation and yield early insights to shape prevention activities addressing health harms associated with opioid and stimulant co-use.

## Supplementary Information

**Additional file 1:** Appendix.

## Data Availability

All posts used in this study were publicly available at the time of collection. Data can be collected via the API mentioned in the article. Aggregated statistics presented in the paper will be shared by the authors upon request.

## Abbreviations

PWUO: People who use opioids
PTMOUD: People being treated with medications for opioid use disorder
OUD: Opioid use disorder
MOUD: Medication for opioid use disorder
NLP: Natural language processing
PWUD: People who use drugs
PWCU: People who co-use (opioids and stimulants)
PWUS: People who use stimulants
API: Application programming interface
