# Supplementary material for: Signals of increasing co-use of stimulants and opioids from online drug forum data
Source: Harm Reduct J. 2022 May 25;19:51. doi: 10.1186/s12954-022-00628-2 (PMC9131693; doi:10.1186/s12954-022-00628-2)
Supplement: Supplementary file 1 — Additional file 1: Appendix. [file 12954_2022_628_MOESM1_ESM.docx]

**Title:** Trends in co-mention of stimulants and opioids: A natural language processing driven analysis of Reddit forums (**Appendix)**

**Authors:**

Abeed Sarker, PhD^1^

Mohammed Ali Al-Garadi, PhD^1^

Yao Ge, MS^1^

Nisha Nataraj, PhD, MS^2^

Christopher M Jones, PharmD, DrPH^2^

Steven A Sumner, MD, MSc^2^

^1^Department of Biomedical Informatics, School of Medicine, Emory University, Atlanta, GA 30322

^2^National Center for Injury Prevention and Control, Centers for Disease Control and Prevention, Atlanta, GA 30341

**Corresponding author:**

Abeed Sarker

101 Woodruff Circle

Suite 4101

Atlanta, GA 30322

Email: abeed@dbmi.emory.edu

**Appendix**

**Table A1.** Names of subreddits included in this study and corresponding numbers of Redditors subscribing to the subreddits at the time

of writing of the manuscript (August, 2021).

| **Subreddit name** | **Number of Redditors subscribing** |
| --- | --- |
| opiates | 127,000 |
| heroin | 32,400 |
| OpiatesRecovery | 32,300 |
| OpiateChurch | 27,800 |
| Methadone | 16,400 |
| suboxone | 14,700 |
| opiates_gonewild | 9,700 |
| fentanyl | 6,800 |
| HeroinHeroines | 4,300 |
| OurOverUsedVeins | 3,200 |
| opiateswriters | 2,400 |
| Carfentanil | 481 |
| Vivitrol | 303 |
| InAHeroinSunrise | <100; [currently removed] |

**Table A2.** Substance names and their automatically-generated misspellings.

| **OPIOIDS**  ***Prescription Opioids***  **hydrocodone** hyrdrocodone hydrocodiene hydro_codone hydrocodene hydrocordone hydorcodone hydrocodeine hidrocodone hyrocodone hydrocone hydrodone hyrdocodone hydrcodone hydrocodones hydrocordisone hydocodone hydrocode hydrocodons hydracodone hydrocodone hydrocodin hydrocodine hydrocodon hydrocondone hydrocodne hydrocdone hydros 357s lortab lortabs lorcet lorcets lortab norco **vicodin** vicoden vicadin viodin vicodin vicodines vicodan vicodien viccodin vocodin vicondin vicoding vicodins vicodon vicidin vidodin vikodin viacodin vicodine vicdin vicotin **percocet** perkocet percocete percacet pecocet percocette perocets percoets percoet percot perocet percoset percocets percocett pecocets percocoet percocit percet percoct percocet10 percicet percocetes percecet percocet **oxycodone** oxocodone oxycodene oxycondone oycodone oxyxodone oxycodones oxicodone oxy_codone oxycodone oxycodine roxycodone ocycodone oxycodons oxcodone oycondone oxycodon oyxcodone oxcycodone oxycodne oxy oxys roxy roxies roxicodone OC OP percs M30 M30s dilaudid hydromorphone oxymorphone **oxycontin** ocycontin oxcontin oxcotin oxcycontin oxycotine oxycontin roxycontin oycotin oxyconton oxycontine oxycotins oxycontin oxycintin oxy_contin oxicontin oxycontins oxycottin oycontin  **morphine** morphin morfin morphs  **tramadol** tramadol trmadol tramdol tramadol  ***Illicit Opioids***  **fentanyl** fentinyl fentenyl fenanyl fentanly fentnyal fentanol fental fetanyl fentayl fentanayl fentanyl fentyl fentanal fetnyl fentynyl fentnayl fentanl fentyanl fentonyl fentanyal fentany fentnyl fent fents **carfentanil** carfentanyl carfentanyl carfent  **heroin** heroin herroin herioin heroins    ***Medications for Opioid Use Disorder (MOUD)***  **suboxone** suboxine subuxone suboxne suboxone saboxone seboxone subxone suboxene suboxones soboxone suboxon subs subutex zubsolv bunavail probuphine **sublocade** sublocade **methadone** methodone methadon methadrone methadose methadones methadone mathadone methedone metadone mehtadone methdone **blue_methadone** blue_methadone **buprenorphine** bupenorphine burprenorphine bupronorphine buprenophine bupernorphine buprenorphin buprenorphine bup bupe bupes **naltrexone** naltrexone naltraxone natrexone naltrexon naltroxone vivitrol vivitrol  ***Opioid Reversal Agents***  **naloxone** naloxone nalaxone **narcan** narcan evzio |
| --- |
| **STIMULANTS**  **speedball** speedballin speedballing speedballs speedball **methamphetamine** methamphetamines meth methl meths methi methy crystal_meth crystalmeth crystal ice speed crank crystal_meth crystalmeth meth methl meths meth methy methamphetamine **lisdexamfetamine** lisdexamfetamine dexamfetamine lisdexamphetamine ritalin ritialin ritallin ritaline ritlian ritalan ritatlin ritlin riain retalin ritalins ridalin rittalin rittlin ritalyn rialin ritilin ritalin **vyvanse** vyvans vvyvanse vyvanses vyvanes vyavanse vyvanse17 vyvense vyvannse vyvance vyvanse vanse vyvanss vivanse vyvanze vyvnase vyvase vyvan vyvansse vyvanase vyanese vyvanse” vyvanese vynanse vyanse vvyanse **dextroamphetamine** dextroamphetamines dextroamphetamine levoamphetamine dexamphetamines dextromethamphetamine dexamphetamine **levoamphetamine** dexamphetamine dextroamphetamine levoamphetamine levomethamphetamine  **dexedrine** dexerdrine dexodrine dexetrine dexidrine dexadrine dexedrine amphetamine amphetamine amphetimines amphetemine amphetimine amphetaminesalt **amphetemines** anphetamine amphetamines amphetamine amfetamine **biphetamine** biphetamine **adderall** adderall addrerall sadderall adderell addrall adderals addera adderall adderoll addorall addarall adderrall dadderall aderal adderalll adderallll adderal badderall smadderall addreall adderallxr madderall aderall aderrall adderral adderally adderalls seadderall **goofball** goofballs goofball goof  *Note: Cocaine was excluded from our analyses due to challenges associated with accurately identifying mentions of the drug |

**Table A3.** Negation phrases associated with specific substance keywords.

| stimulant cardiac-stimulant\|cardiac stimulant\|cardiac-stimulant  methamphetamine meth gmt\|meth group\|meth obstet\|meth odist\|meth germantown\|meth gmt\|meth group\|meth obstet  oxycodone not enough oxy\|oxy clean\|oxy deep cleaner\|oxy given\|oxy high\|oxy level\|oxy low\|oxy mask\|pulse oxy |
| --- |

**Table A4.** List of negations used for detecting negated substance mentions.

| no not without absence of  cannot couldn't could not didn't did not denied denies free of negative for never had resolved exclude with no rule out aside from except apart from |
| --- |


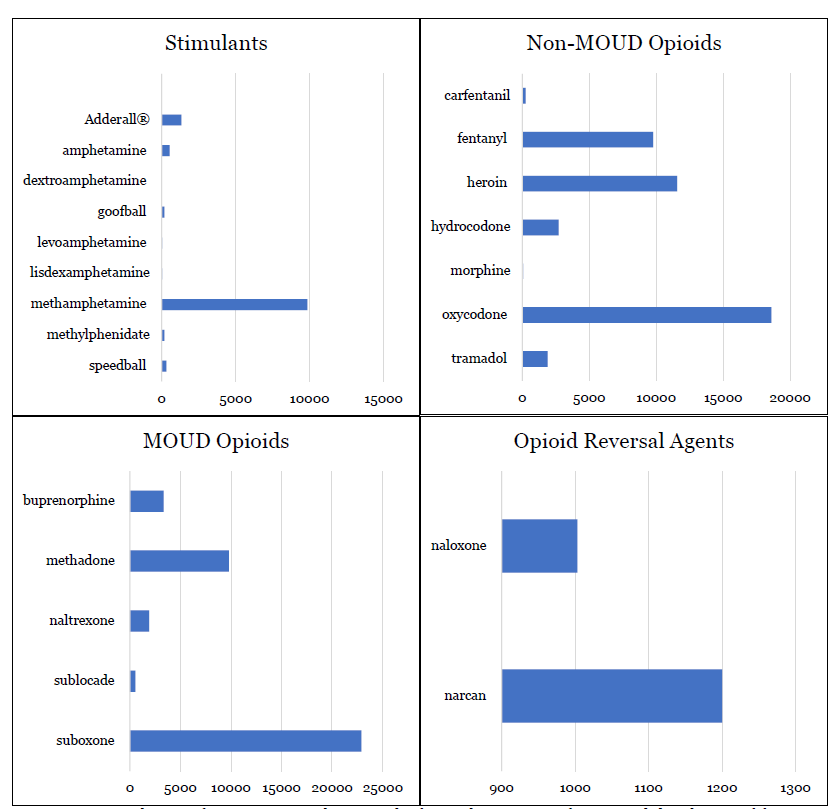


MOUD

Illicit and Prescription Opioids

**Figure A5.** Distribution of mentions per substance, organized alphabetically. The figure shows a more fine-grained distribution of frequencies compared to the groupings used in the article. In the article, the following mentions of substances are grouped together: (i) Speedball/Goofball (*a combination of methamphetamine and heroin*), (ii) levoamphetamine, amphetamine, dextroamphetamine, Adderall® and lisdexamphetamine (*amphetamine type stimulants*), (iii) carfentanil and fentanyl (*fentanyl & analogs*), (iv) morphine, hydrocodone, tramadol and oxycodone (*prescription opioid pain relievers*), (v) naltrexone, sublocade, buprenorphine, methadone and suboxone (*MOUD*), and (vi) narcan and naloxone (*opioid reversal agents*). Heroin and methamphetamine are not grouped with any other keyword/substance.

**Table A6.** Metrics for posts and Redditors from 2011 to 2020.

| **Year** | **Total posts** | **Total Redditors** | **Opioids and opioid-related medications mentions** | **Stimulant mentions** | **People who use opioids or being treated for with medication for opioid use disorder (PWUO/PTMOUD)** | **People who use stimulants (PWUS)** | **People who co-use opioids and stimulants (PWCU)** | **Ratio: PWCU/Redditors** |
| --- | --- | --- | --- | --- | --- | --- | --- | --- |
| 2011 | 9,001 | 244 | 75 | 36 | 22 | 29 | 9 | 0.037 |
| 2012 | 32,152 | 579 | 353 | 132 | 89 | 103 | 35 | 0.060 |
| 2013 | 57,443 | 982 | 885 | 246 | 165 | 195 | 71 | 0.072 |
| 2014 | 84,697 | 1,440 | 1,284 | 326 | 243 | 263 | 98 | 0.068 |
| 2015 | 141,044 | 1,959 | 1,756 | 474 | 381 | 370 | 156 | 0.080 |
| 2016 | 212,145 | 2,898 | 3,529 | 750 | 619 | 578 | 261 | 0.090 |
| 2017 | 398,217 | 4,474 | 6,852 | 1,298 | 1,132 | 997 | 523 | 0.117 |
| 2018 | 772,258 | 6,713 | 13,507 | 2,166 | 2,053 | 1,660 | 910 | 0.136 |
| 2019 | 1,306,354 | 9,128 | 20,624 | 3,021 | 2,944 | 2,351 | 1,288 | 0.141 |
| 2020 | 2,140,854 | 11,072 | 31,417 | 3,669 | 3,930 | 2,866 | 1,718 | 0.155 |
